# The role of PD-1 in regulation of macrophage apoptosis and its subversion by *Leishmania donovani*

**DOI:** 10.1038/cti.2017.12

**Published:** 2017-05-05

**Authors:** Shalini Roy, Purnima Gupta, Shreyasi Palit, Moumita Basu, Anindita Ukil, Pijush K Das

**Affiliations:** 1 Infectious Diseases and Immunology Division, CSIR-Indian Institute of Chemical Biology, Kolkata, India; 2 Department of Biochemistry, Calcutta University, Kolkata, India

## Abstract

Programmed death-1 receptor (PD-1) expressed in many immune cells is known to trigger T-cell exhaustion but the significance of macrophage-associated PD-1 in relevance to macrophage apoptosis is not known. This study is aimed to delineate whether PD-1 pathway has any role in eliciting macrophage apoptosis and, if so, then how the intra-macrophage parasite, *Leishmania donovani* modulates PD-1 pathway for protecting its niche. Resting macrophages when treated with H_2_O_2_ showed increased PD-1 expression and apoptosis, which was further enhanced on PD-1 agonist treatment. The administration of either PD-1 receptor or PD-1 ligand-blocking antibodies reversed the process thus documenting the involvement of PD-1 in macrophage apoptosis. On the contrary, *L. donovani*-infected macrophages showed decreased PD-1 expression concurrent with inhibition of apoptosis. The activation of PD-1 pathway was found to negatively regulate the phosphorylation of pro-survival AKT, which was reversed during infection. Infection-induced PD-1 downregulation led to the activation of AKT resulting in phosphorylation and subsequent inhibition of proapoptotic protein BAD. Strong association of SHP2 (a SH2-containing ubiquitously expressed tyrosine-specific protein phosphatase) with PD-1 along with AKT deactivation observed in H_2_O_2_-treated macrophages was reversed by *L. donovani* infection. Kinetic analysis coupled with inhibitor-based approach and knockdown experiments demonstrated that *L*. *donovani* infection actively downregulated the PD-1 by deactivating NFATc1 as revealed by its reduced nuclear translocation. The study thus elucidates the detailed mechanism of the role of PD-1 in macrophage apoptosis and its negative modulation by *Leishmania* for their intracellular survival.

Programmed cell death-1 receptor (PD-1), a type I transmembrane glycoprotein, belongs to the CD28 family and is known to mediate apoptosis in T cells either as a measure of tumor invasion^1, 2, 3, 4^ or in chronic infections.^5, 6, 7, 8, 9^ Although PD-1 shares 33% sequence identity with CTLA-4, another co-inhibitory receptor,^2^ the expression of CTLA-4 is restricted only to T cells, whereas the expression of PD-1 can be induced in diverse immune cell types comprising of T cells, B cells and myeloid cells, implying a much broader role of PD-1 in immune regulation.^1, 4^ PD-1:PD-1L (PD-1 ligand) pathway has a pivotal role in the inhibition of T-cell function through the engagement of PD-1 on T cells with its ligands PD-1L1 or PD-1L2 present on antigen-presenting cells. Blockade of PD-1 or genetic ablation of PD-1L1 or PD-1L2 on dendritic cells or other antigen-presenting cells enhances their capacity to stimulate T-cell responses *in vitro* as compared with wild-type antigen-presenting cells.^10, 11^ PD-1 was initially cloned as a molecule that was found to be overexpressed in apoptotic cells.^3^ However, recent emerging evidences also highlight the significance of this pathway in triggering immunosuppression in the context of various diseases including cancer^12, 13^ and infectious diseases.^5, 10, 14, 15^ PD-1 has two motifs ITIM (immunoreceptor tyrosine-based inhibitory motif) and ITSM (immunoreceptor tyrosine-based switch motif) of which the recruitment of SH2-containing tyrosine phosphatases SHP1 and SHP2 to ITSM inhibits PD-1 functions.^1, 2^ Because of stronger interaction of SHP2, PD-1 is likely to recruit SHP2 in mediating dephosphorylation of AKT.^1, 2, 16^ PD-1 pathway is known to have a critical role in mediating T-cell exhaustion in many chronic infections including leishmaniasis.^6, 7, 8, 15, 17^ Majority of these studies have well appreciated the importance of PD-1 pathway in triggering T-cell apoptosis thereby abrogating host-defense and facilitating disease progression. However, although PD-1 is constitutively expressed in macrophages,^18^ the physiological role of macrophage-associated PD-1 is yet to be elucidated.

AKT signaling is known to have a role in cell growth and survival acting as a major pro-survival pathway,^19^ and earlier studies demonstrated that *L. donovani* infection induces activation of AKT pathway to prevent host-cell death.^20, 21^ In the present study it has been demonstrated that PD-1/SHP2 signaling axis negatively regulates pro-survival AKT activation in macrophages and H_2_O_2_-mediated PD-1 induction contributes in macrophage apoptosis. Besides, as inhibition of macrophage apoptosis is critically important in leishmaniasis^22, 23, 24, 25, 26^ and is an established paradigm of infection, therefore, modulation of death receptor PD-1 expression during infection might have an impact in regulating host-cell apoptosis, which also is addressed here. Our findings further shed light on the fact that *L. donovani* infection-mediated downregulation of PD-1 and subsequent dampening of PD-1/SHP2 signaling helped the parasite in activating pro-survival AKT pathway, which in turn led to inhibition of proapoptotic protein BAD, thereby inhibiting host-cell apoptosis.

## Results

### Role of PD-1 in macrophage apoptosis

**Figure 1:**
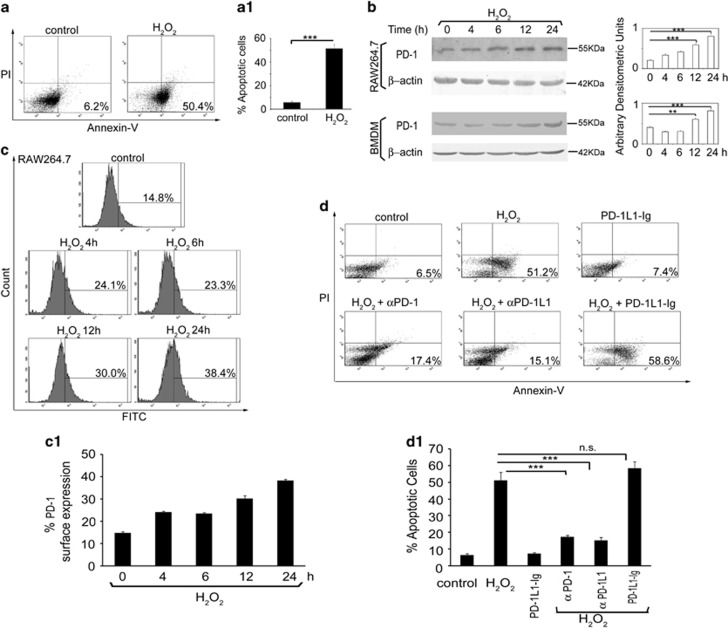
Role of PD-1 in macrophage apoptosis. (**a**) RAW 264.7 macrophages were treated with H_2_O_2_ (400 μm) for 1 h. The cells were washed, incubated overnight at 37 °C and the extent of apoptosis was analyzed by annexin-V-tagged FITC-PI flow cytometry. Dual parameter dot plot of FITC fluorescence (*x* axis) versus PI fluorescence (*y* axis) is represented as logarithmic fluorescence intensity. Quadrants: upper left, necrotic cells; lower left, live cells; lower right, apoptotic cells; upper right, necrotic or late phase of apoptotic cells. (**a1**) Individual bar graph represents the mean percentage±s.d. of apoptotic cells. (**b**, **c**) RAW 264.7 or BMDM were treated with H_2_O_2_ (400 μm) for 1 h. The cells were washed and collected at the indicated time points post H_2_O_2_ treatment and the expression of PD-1 was monitored by immunoblotting (**b**) and flow cytometry (**c**). (**c1**) Individual graph denotes the mean percentage±s.d. of cells expressing PD-1. (**d**) RAW 264.7 macrophages were treated either with anti-PD-1 or anti-PD-1L1 antibody or PD-1 agonist (PD-1L1-Ig fusion protein) followed by treatment with H_2_O_2_ and the extent of apoptosis was analyzed by annexin-V-PI flow cytometry. (**d1**) Individual bar graph represents the mean percentage±s.d. of apoptotic cells. Bands were analyzed densitometrically and expressed as mean±s.d; *n*=3, NS, nonsignificant; **P<0.01, ***P<0.001; Student’s *t*-test. PI, propidium iodide.

Role of PD-1 in regulating T-cell apoptosis is well documented. However, although macrophages express both PD-1 receptor and ligand, no work has yet been done on the involvement of PD-1 signaling in inducing macrophage apoptosis,^8, 10, 11, 12, 13, 14, 15^ Therefore, we thought it worthwhile to study the role of PD-1 pathway in macrophage apoptosis. We treated RAW 264.7 cells with the apoptotic inducer H_2_O_2_ (400 μm) for 1 h and determined the percentage of annexin-positive cells after overnight incubation by FACS analysis (Figure 1a) and time-dependent PD-1 expression by western blot analysis (Figure 1b). Along with the increase in apoptosis (50.4±5.1% as compared with 6.2±0.7% in control cells *P*<0.001; Figure 1a), H_2_O_2_ treatment resulted in higher PD-1 level as observed upto 24 h with maximum at 24 h post treatment (3.9-fold compared with untreated macrophages, *P*<0.001; Figure 1b). We further validated these findings in BMDM, which also depicted increased PD-1 expression at 12 and 24 h post treatment (Figure 1b). Western blot data were corroborated by flow cytometry analysis, which showed maximum surface expression of PD-1 at 24 h post H_2_O_2_ treatment (Figure 1c). To further ascertain the role of PD-1 in macrophage apoptosis, RAW 264.7 cells were pretreated with monoclonal antibodies against either PD-1 or PD-1 ligand (PD-1L1) and then subjected to H_2_O_2_ treatment (Figure 1d). Flow cytometry analysis revealed that as compared with H_2_O_2_-treated cells, which showed 51.2±4.7% apoptosis, PD-1- and PD-1L1-blocked macrophages showed significantly decreased apoptosis (17.4±0.9% and 15.1±1.8%, respectively, *P*<0.001) thereby emphasizing the role of PD-1 signaling in macrophage apoptosis. In contrast, the PD-1 agonist (PD-1L1-Ig fusion protein) pretreated and H_2_O_2_-administered cells showed slight increase in apoptosis (58.6±3.9% compared with 51.2±4.7% in H_2_O_2_-treated cells, *P*>0.05; Figure 1d). Taken together, these results suggest that macrophage apoptosis is associated with increased PD-1 expression and blockade of PD-1 signaling reduced macrophage apoptosis.

### Role of PD-1 in *Leishmania*-induced inhibition of macrophage apoptosis

**Figure 2:**
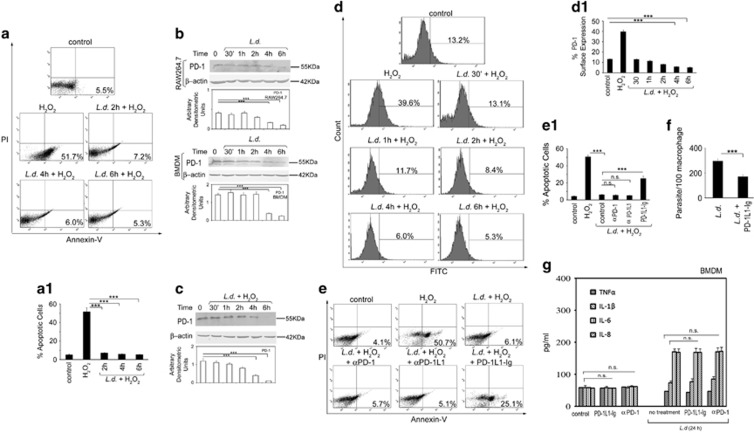
Role of PD-1 in *Leishmania*-induced inhibition of macrophage apoptosis. (**a**) RAW 264.7 cells were infected with *L. donovani* (L.d.) promastigotes with a parasite/macrophage ratio of 10:1 for the indicated time periods (0–6 h) followed by treatment with H_2_O_2_ (400 μm) for 1 h. The cells were washed and incubated overnight at 37 °C and the extent of apoptosis was analyzed by annexin-V-tagged FITC-PI flow cytometry. (**a1**) Individual bar graph represents the mean percentage±s.d. of apoptotic cells. (**b**) Both RAW 264.7 and BMDM cells were infected with promastigotes of *L. donovani* (L.d) for the indicated time periods (0–6 h) and expression of PD-1 was evaluated at the protein level by immunoblotting. (**c**, **d**) RAW cells were infected with *L. donovani* (L.d.) for the indicated time period followed by treatment with H_2_O_2_ (400 μm) for 1 h. Expression of PD-1 was analyzed by western blotting (**c**) and flow cytometry (**d**). (**d1**) Individual graph represents the mean percentage±s.d. of cells expressing PD-1. (**e**) RAW cells were pretreated with either anti-PD-1 or anti-PD-1L1 antibody or PD-1L1-Ig and then infected with *L. donovani* promastigotes for 6 h followed by treatment with H_2_O_2_ (400 μm) for 1 h. The extent of apoptosis was analyzed by flow cytometry. (**e1**) Individual bar graph represents the mean percentage±s.d. of apoptotic cells. (**f**) RAW cells were pretreated with PD-1 agonist PD-1L1-Ig followed by infection with *L. donovani* promastigotes for 4 h and intracellular parasite number was determined by Giemsa staining after 24 h. (**g**) BMDM were pretreated with either anti-PD-1 antibody or PD-1L1-Ig and infected with *L. donovani* promastigotes. Supernatants were collected at 24 h post infection and levels of cytokines TNFα, IL-1β, IL-6 and CXCL8 or IL-8 in the cell supernatants were determined by ELISA. Results are representative of three independent experiments performed in triplicate. The data shown are mean±s.d.; *n*=3, NS, nonsignificant; ***P<0.001; Student’s *t*-test.

Inhibition of host-cell apoptosis is one of the survival strategies used by intracellular pathogens.^27, 28, 29^ *L. donovani,* an intra-macrophage parasite, also exploits various mechanisms to inhibit host-cell apoptosis.^19, 20, 21, 22, 23^ To determine whether *Leishmania* parasite modulates PD-1 pathway for inhibition of macrophage apoptosis, we checked the effect of *L.donovani* infection on macrophage apoptosis and its correlation with PD-1 receptor. Flow cytometry analysis revealed that*L. donovani* infection significantly decreased H_2_O_2_-induced macrophage apoptosis (7.2±0.5, 6.0±0.5, 5.3±0.6% at 2, 4 and 6 h post infection, respectively compared with 51.7±4.4% in H_2_O_2_-treated control; Figure 2a). We then determined the kinetics of PD-1 expression in *L.donovani*-infected RAW 264.7 macrophages, which decreased gradually with a maximum reduction obtained at 6 h post infection (84.2% reduction compared with uninfected cells, *P*<0.001, Figure 2b) indicating infection-induced inhibition of PD-1 expression. Similar observation was noted in BMDM (Figure 2b, lower panel). As PD-1 was found to be induced upon H_2_O_2_ treatment, we therefore wanted to determine whether *L. donovani* infection can modulate the PD-1 expression in H_2_O_2_-treated macrophages. To this end, macrophages were infected with *L. donovani* promastigotes followed by treatment with H_2_O_2_, and PD-1 expression was determined after indicated time periods. Like infected cells, PD-1 level was also reduced in infected H_2_O_2_-treated cells (66.2 and 91.6% reduction at 4 and 6 h post infection, respectively, compared with untreated cells, *P*<0.001; Figure 2c). Furthermore, flow cytometry analysis also showed similar trend in the reduction in surface expression of PD-1 in *L. donovani*-infected H_2_O_2_-treated RAW 264.7 macrophages (55.3 and 60.6% reduction at 4 and 6 h post infection, respectively, compared with untreated cells, *P*<0.001; Figure 2d). We also showed that compared with H_2_O_2_-treated cells (50.7±1.8% apoptosis), there was significant reduction in apoptosis in the infected cells (6.1±0.4% apoptosis), which was not further altered by treatment with anti-PD-1 or anti-PD-1L1 antibody (5.7±0.4 and 5.1±0.4, *P*>0.05, respectively), however, a little increase in the agonist-pretreated infected cells (25.1±2.7%, *P*<0.001) was observed suggesting thereby that infection has already inhibited PD-1-mediated signaling (Figure 2e). As shown in Figure 2f, PD-1 agonist treatment significantly reduced parasite burden (42.4% reduction compared with infected control, *P*<0.001) thereby implicating the importance of PD-1 in *Leishmania* infection. The contribution of PD-1 pathway in negatively regulating M1 polarization of macrophages^30^ suggested an anti-inflammatory influence of PD-1 in macrophages. Therefore, we thought it worthwhile to ascertain whether PD-1 signaling has any impact upon the levels of pro-inflammatory cytokines TNFα, IL-1β, IL-6 and CXCL8 or IL-8 both in control and infected macrophages. We measured the levels of the cytokines by ELISA in the culture supernatants of uninfected and infected (24 h) BMDM treated with either PD-1 agonist (PD-1L1-Ig fusion protein, 2.5 μg ml^−1^) or antagonistic anti-PD-1 antibody (10 μg ml^−1^). Although the levels of pro-inflammatory cytokines were very low in uninfected macrophages, *L. donovani* infection resulted in moderate increase in the levels of IL-6 and IL-8 (169.13±10.6 pg ml^−1^ and 168.27±10.5 pg ml^−1^ of IL-6 and IL-8) in infected BMDM compared with 57.3±0.5 pg ml^−1^ of IL-6 and 56.7±0.7 pg ml^−1^ of IL-8 in the control BMDM, respectively (Figure 2g). However, no detectable difference was observed in levels of cytokines in PD-1 agonist or in PD-1 antagonist-treated control or infected macrophages compared with untreated control (Figure 2g). This might be due to the downregulation of PD-1 receptor during *L. donovani* infection. Collectively, these results revealed that *L. donovani* inhibits macrophage apoptosis by reducing PD-1 expression thereby inhibiting PD-1 signaling.

### Role of PD-1 pathway on activation of AKT

**Figure 3:**
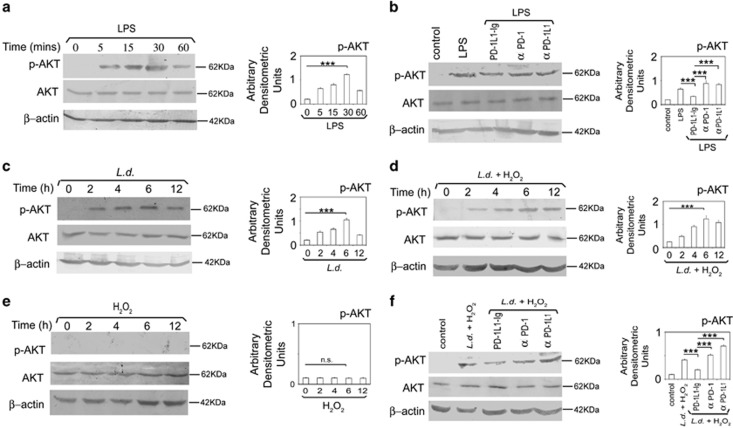
Role of PD-1 pathway on activation of AKT. (**a**) RAW cells were treated with LPS (100 ng ml^−1^) for indicated time period. Expressions of phospho-AKT and total AKT were analyzed by immunoblotting. (**b**) RAW cells were pretreated with either PD-1L1-Ig or anti-PD-1 antibody or anti-PD-1L1 antibody followed by LPS (100 ng ml^−1^) treatment for 30 min. The cell lysates were analyzed by western blotting with indicated antibodies. (**c**–**e**) RAW cells were infected with *L. donovani* promastigotes for indicated time periods followed by treatment with H_2_O_2_ for 1 h. The cells were then analyzed for the level of p-AKT and AKT by western blotting in infected cells (**c**), infected and H_2_O_2_-treated cells (**d**) and H_2_O_2_-treated cells (**e**). (**f**) RAW cells were pretreated with either anti-PD-1 antibody or anti-PD-1L1 antibody or PD-1L1-Ig and then infected with *L. donovani* promastigotes for 6 h followed by treatment with H_2_O_2_ for 1 h. The cells were analyzed for the level of p-AKT and AKT by western blotting. The data shown are representative of three independent experiments. Bands were analyzed densitometrically and expressed as mean±s.d.; *n*=3, NS, nonsignificant; ***P<0.001; Student’s *t*-test.

To examine whether PD-1-mediated apoptosis has any influence on AKT signaling, which is associated with cell growth and survival,^19^ we assessed the impact of PD-1 agonist-mediated increased PD-1 signaling on LPS-induced activation of AKT in the macrophages. Maximum AKT phosphorylation (6.2-fold increase compared with control, *P*<0.001, Figure 3a) was observed post 30 min LPS (100 ng ml^−1^) treatment (Figure 3a) and 30 min time point was therefore chosen in all the subsequent experiments involving LPS treatment. PD-1 agonist-pretreated RAW 264.7 macrophages showed considerable decline in LPS-mediated AKT phosphorylation (47.2% reduction compared with only LPS-treated macrophages, *P*<0.001, Figure 3b) suggesting that PD-1 signaling may antagonize AKT activation. To ascertain the negative regulation of AKT by PD-1 pathway, we next evaluated the effects of PD-1 pathway blockade on AKT activation. Contrary to PD-1 agonist, LPS-mediated AKT phosphorylation was significantly enhanced when PD-1 signaling was inhibited by using anti-PD-1 or anti-PD-1L1 antibody (2.6- and 2.5-fold, respectively, compared with agonist-treated cells, *P*<0.001; Figure 3b). Since *Leishmania* parasite induces activation of AKT pathway for inhibition of host-cell apoptosis,^20^ we wanted to determine whether *Leishmania* downregulates PD-1 to activate AKT pathway. Kinetic analysis revealed that infection resulted in increased phosphorylation of AKT as observed upto 12 h with a maximum at 6 h post infection (5.0-fold increase compared with control, *P*<0.001; Figure 3c). Similar trend was observed in infected RAW 264.7 macrophages treated with H_2_O_2_ (5.1-fold increase compared with control at 6 h post infection, *P*<0.001; Figure 3d). However, RAW 264.7 macrophages treated with H_2_O_2_ alone did not result in p-AKT activation (Figure 3e). Next, we wanted to assess the impact of PD-1 pathway on *Leishmania*-induced AKT phosphorylation. Phospho-AKT level was markedly reduced in agonist-pretreated infected RAW 264.7 macrophages (51.2% reduction over infected control, *P*<0.001) whereas it was significantly increased in macrophages pretreated either with anti-PD-1 or anti-PD-1L1 antibodies (3.2- and 4.3-fold, respectively, over agonist-treated infected cells, *P*<0.001, respectively, Figure 3f) thereby suggesting the reciprocal relationship between AKT and PD-1 signaling during infection. Taken together, these results suggest that PD-1 pathway negatively regulates AKT activation and downregulation of PD-1 by *L. donovani* infection led to the activation of AKT.

### Role of PD-1/SHP2 signaling axis in regulation of AKT

**Figure 4:**
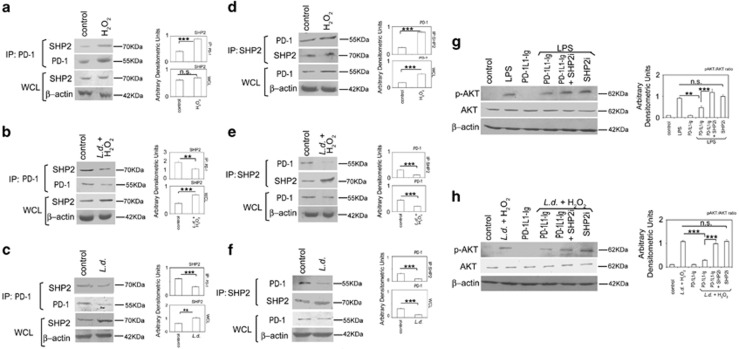
The role of PD-1/SHP2 signaling axis on activation of AKT. (**a**–**c**) RAW 264.7 macrophages infected with *L. donovani* promastigotes for 6 h or control RAW 264.7 cells were treated with H_2_O_2_ (400 μm) for 1 h, washed and kept overnight at 37 °C. The cell lysates were then immunoprecipitated with anti-PD-1 antibody and analyzed for the expression of SHP2 by immunoblotting in H_2_O_2_-treated cells (**a**), infected and H_2_O_2_-treated cells (**b**) and infected cells (**c**). Level of SHP2 in the whole-cell lysate (WCL) was analyzed by immunoblotting using anti-SHP2 antibody in **a**–**c**. (**d**–**f**) *L. donovani*-infected (6 h) macrophages or uninfected control macrophages were treated with H_2_O_2_ for 1 h, washed and kept overnight at 37 °C. Lysates were immunoprecipitated using anti-SHP2 antibody and analyzed for the expression of PD-1 by immunoblotting in H_2_O_2_-treated cells (**d**), infected and H_2_O_2_-treated cells (**e**) and infected cells (**f**). Level of PD-1 in the whole-cell lysate (WCL) was analyzed by immunoblotting using anti-PD-1 antibody in **d**–**f**. (**g**) RAW cells were treated with either LPS (100 ng ml^−1^ for 30 min) or PD-1 agonist PD-1L1-Ig (2.5 μg μl^−1^ for 2 h) or with both in the presence or absence of SHP2 inhibitor, orthovanadate (200 μm for 45 min). The cell lysates were analyzed for the expression of p-AKT and total AKT by western blot analysis. (**h**) PD-1 agonist-pretreated macrophages were infected with *L*. *donovani* promastigotes for 6 h followed by treatment with H_2_O_2_ for 1 h in the presence or absence of SHP2 inhibitor. The cell lysates were then analyzed for p-AKT and AKT levels by western blot analysis.The data shown are one of three independent experiments. Bands were analyzed densitometrically and expressed as mean±s.d.; *n*=3, NS, nonsignificant; **P<0.01; ***P<0.001; Student’s *t*-test.

As PD-1:PD-IL engagement induces recruitment of SHP2 in the ITSM domain present in cytoplasmic tail of PD-1,^1, 2^ we wanted to determine whether PD-1 uses SHP2 as its downstream effector in mediating AKT deactivation. We studied the interaction between PD-1 and SHP2, both in control and H_2_O_2_-treated RAW macrophages by co-immunoprecipitation studies, which revealed strong association (2.2-fold compared with control, *P*<0.001) of PD-1 with SHP2 in H_2_O_2_-treated macrophages (Figure 4a). However, although an increase in PD-1 level was observed in H_2_O_2_-treated immunoprecipitated samples, SHP2 level was found to be same in both control and H_2_O_2_-treated cells (Figure 4a). To verify our observation, reciprocal co-immunoprecipitation was performed where SHP2 was immunoprecipitated followed by immunoblotting with PD-1. Significantly increased association of PD-1 with immunoprecipitated SHP2 (3.3-fold compared with control, *P*<0.001, Figure 4d) conformed to our earlier observation suggesting thereby that increased PD-1 expression probably leads to its increased association with SHP2. Next, to ascertain the role of SHP2 in AKT phosphorylation, we studied induction of AKT phosphorylation by LPS in PD-1 agonist-pretreated macrophages in the presence and absence of SHP2 inhibitor. Phospho-AKT levels were markedly diminished in RAW 264.7 macrophages pretreated with LPS and PD-1 agonist (46.2% decrease compared with LPS-treated control, *P*<0.01), which was reversed in the presence of SHP2 inhibitor (2.6-fold increase, *P*<0.001, Figure 4g). However, the level of LPS-induced AKT phosphorylation in the presence of SHP2 inhibitor alone was quite similar to that observed in PD-1 agonist and SHP2 inhibitor co-treated macrophages (Figure 4g). We then wanted to study the status of PD-1/SHP2 signaling axis in the context of *L. donovani* infection. PD-1 was immunoprecipitated from H_2_O_2_-treated infected macrophages and subjected to western blotting with anti-SHP2 antibody, which revealed decreased association of PD-1 with SHP2 (40.1% reduction compared with control, *P*<0.01; Figure 4b). Endogenous SHP2 was markedly increased (1.8-fold compared with control, *P*<0.001) in whole-cell lysates of infected cells (Figure 4b). Reciprocal co-immunoprecipitation studies where SHP2 was immunoprecipitated followed by immunoblotting with PD-1 also showed significantly decreased association of PD-1 with SHP2 (65.2% compared with control, *P*<0.01; Figure 4e) while the endogenous PD-1 in whole-cell lysate was also significantly reduced in infected cells (53.8% compared with control, *P*<0.001; Figure 4e). Identical experiments carried out with *L.donovani*-infected RAW 264.7 macrophages without post H_2_O_2_ treatment, also documented similar findings as above (Figures 4c and f). These results suggest that decreased association of PD-1 with SHP2 is a consequence of PD-1 downregulation during infection. Next, to delineate the role of SHP2 as a downstream mediator of PD-1- mediated AKT deactivation, infected and PD-1 agonist-pretreated RAW 264.7 macrophages were subjected to H_2_O_2_ treatment in the presence or absence of SHP2 inhibitor. PD-1 agonist treatment resulted in decline of infection-induced increase of phospho-AKT level (73.5% decrease compared with infected control, *P*<0.001), which was rescued in the presence of SHP2 inhibitor (Figure 4h). To validate further the role of SHP2, we used an *in vitro* siRNA knockdown system for SHP2, which also demonstrated that SHP2 knockdown appreciably rescued the decline in AKT phosphorylation occurring in the agonist-treated infected cells (Supplementary Figure 1). The efficiency of knockdown was determined by western blotting, which showed 83.6% (Supplementary Figure 2).

### Role of PD-1 pathway on proapoptotic factors in macrophages

**Figure 5:**
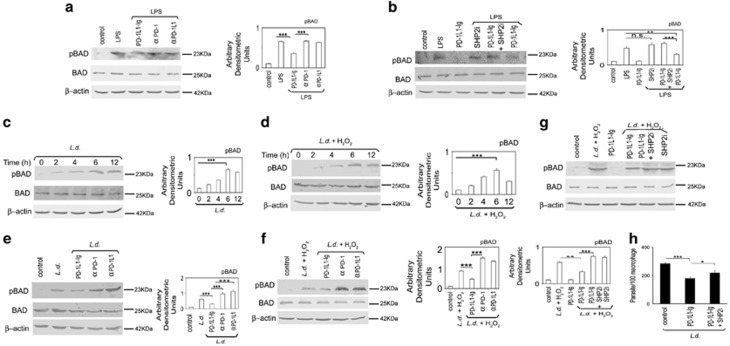
Role of PD-1 pathway on proapoptotic factors in macrophages. (**a**) RAW cells were pretreated either with anti-PD-1 or anti-PD-1L1 antibody or PD-1 agonist (PD-1L1-Ig) followed by treatment with LPS (100 ng ml^−1^ for 30 min). The cell lysates were analyzed for the expression of phospho-BAD and BAD by western blotting using respective antibodies. (**b**) RAW cells were treated with PD-1 agonist or SHP2 inhibitor, sodium orthovanadate or both followed by treatment with LPS (100 ng ml^−1^ for 30 min). The cell lysates were analyzed for the levels of p-BAD and BAD. (**c**, **d**) The cells were infected with *L. donovani* promastigotes for indicated time periods and treated with or without H_2_O_2_ (400 μm) for 1 h. The cell lysates were analyzed for p-BAD and BAD in infected (**c**) and infected and H_2_O_2_-treated (**d**) cells. (**e**, **f**) *L. donovani*-infected (6 h) macrophages were pretreated either with anti-PD-1 or anti-PD-1L1 antibody or PD-1 agonist followed by treatment with or without H_2_O_2_ (400 μm) for 1 h. THe cell lysates were analyzed for p-BAD and BAD in infected (**e**) and infected and H_2_O_2_-treated (**f**) cells. (**g**) The cells were treated with PD-1 agonist or SHP2 inhibitor, sodium orthovanadate or both, infected with *L. donovani* promastigotes and treated with H_2_O_2_. p-BAD and BAD levels were analyzed by immunoblotting. (**h**) The cells were pretreated with PD-1 agonist in the presence or absence of SHP2 inhibitor, infected with *L. donovani* promastigotes for 4 h and intracellular parasite number was determined by Giemsa staining after 24 h. The data shown are mean±s.d. of three independent experiments; *n*=3, NS, nonsignificant; *P<0.05; **P<0.01; ***P<0.001; Student’s *t*-test.

AKT pathway has an important role in cell survival through activation and inactivation of anti- and proapoptotic proteins, respectively, by phosphorylation/dephosphorylation.^31, 32, 33^ BAD is a well -characterized proapoptotic protein and AKT-mediated phosphorylation of BAD renders it inactive.^32^ PD-1 pathway negatively regulates pro-survival AKT, therefore, we wanted to delineate its impact on proapoptotic BAD. To this end, RAW 264.7 macrophages were analyzed for BAD phosphorylation in response to PD-1 agonist or antagonist using LPS as positive control. Phospho-BAD level was significantly decreased in PD-1 agonist-treated macrophages (48.4% decrease compared with LPS-treated macrophages, *P*<0.01) whereas PD-1 antagonist (anti-PD-1 or anti-PD-1L1 antibody)-treated cells showed 2.9- and 3.4-fold increase (*P*<0.001) over agonist-treated macrophages (Figure 5a). To ascertain whether SHP2 works downstream of PD-1 in mediating the negative regulation of BAD phosphorylation, phospho-BAD levels were analyzed in agonist-pretreated and LPS-administered cells in the presence or absence of SHP2 inhibitor. Decreased BAD phosphorylation in PD-1 agonist-pretreated RAW 264.7 macrophages could be partially reversed by SHP2 inhibition (2.1-fold increase, *P*<0.001, Figure 5b). In case of *L. donovani* infection, BAD phosphorylation was found to be increased in infected as well as infected H_2_O_2_-treated RAW 264.7 macrophages with a maximum at 6 h post infection (5.6- and 5.5-fold increase over control, *P*<0.001, Figures 5c and d). Increased BAD phosphorylation in *L. donovani*-infected H_2_O_2_-treated macrophages was found to be inhibited by PD-1 agonist (45.8% decrease compared with infected control, *P*<0.001), whereas PD-1 antagonist (anti-PD-1 or anti-PD-1L1 antibody) treatment resulted in an increase in BAD phosphorylation (3.2- and 2.9-fold increase over agonist-treated infected cells, *P*<0.001, Figure 5f). Similar findings were obtained in identical experimental set using only infected RAW 264.7 macrophages without H_2_O_2_ treatment (Figure 5e). In *L. donovani* infection also, BAD phosphorylation is mediated by SHP2, as the decrease in infection-induced BAD phosphorylation by agonist treatment could be partially reversed by using SHP2 inhibitor (2.3-fold increase, *P*<0.001, Figure 5g). Similar results were obtained when we used SHP2 siRNA instead of SHP2 inhibitor (Supplementary Figure 3). This was also reflected in parasite survival as the reduced intra-macrophage survival by PD-1 agonist treatment (37.1% reduction compared with infected control) could be partially reversed by treatment with SHP2 inhibitor but only a slight decrease in parasite suppression was observed in infected cells treated with PD-1 agonist along with SHP2 inhibitor (23.1% reduction compared with infected control; Figure 5h).

### Role of NFATc1 in *L. donovani* infection-induced PD-1 down regulation

**Figure 6:**
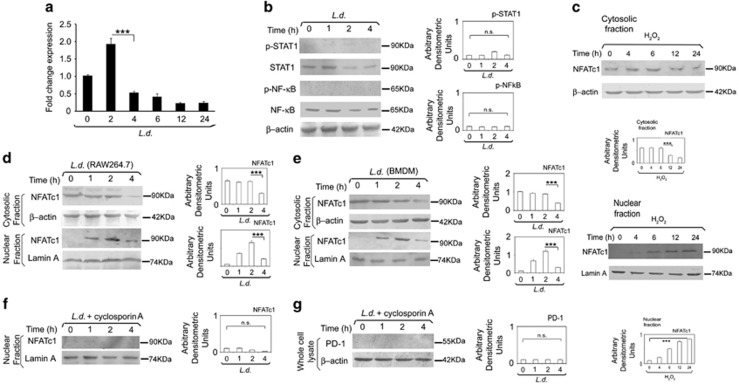
Role of NFATc1 in *L. donovani* infection-induced PD-1 downregulation. (**a**) RAW 264.7 macrophages were infected with *L. donovani* promastigotes for various time periods as indicated and the expression of PD-1 was evaluated at the mRNA level by real-time PCR. (**b**) RAW cells were infected with *L. donovani* promastigotes at indicated times and phospho-STAT1, STAT1, phospho-P65 NF-κB and P65 NF-κB levels were analyzed by western blotting. (**c**) RAW cells were treated with H_2_O_2_ (400 μm) for 1 h. The cells were washed and kept overnight at 37 °C. Levels of NFATc1 were analyzed in nuclear and cytosolic fraction by western blot using anti-NFATc1 antibody. Lamin A and β-actin were used as internal control for nuclear and cytosolic fraction, respectively. (**d**, **e**) Macrophages were infected with *L. donovani* promastigotes at indicated time periods and the levels of NFATc1 was analyzed in nuclear and cytosolic fractions by western blot in RAW (**d**) and BMDM (**e**). (**f**, **g**) RAW cells were treated with cyclosporine A (1 μg ml^−1^) for 24 h followed by infection with *L. donovani* promastigotes for the indicated times. Level of NFATc1 was then analyzed in nuclear fraction (**f**) and the level of PD-1 was analyzed in whole-cell lysate (**g**) by western blotting. The data shown are mean±s.d. of three independent experiments; *n*=3, NS, nonsignificant; ***P<0.001; Student’s *t*-test.

To get an insight into the molecular mechanism of PD-1 down regulation by *L. donovani* infection, we studied the kinetics of PD-1 mRNA level in infected cells by real-time PCR analysis, which was decreased substantially from 4 h time point as observed upto 12 h post infection (Figure 6a). As *L. donovani* infection resulted in repression of PD-1, we looked for transcription factors possibly mediating the repression. PD-1 is reported to be transcriptionally regulated by STAT1 and NF-κB.^18, 34^ We therefore studied activation kinetics of these two transcription factors in infected cells. However, neither STAT1 nor NF-κB p65 showed any activation upto 4 h post infection (Figure 6b), suggesting that these two transcription factors may not be responsible for maintaining initial PD-1 level during infection. As nuclear factor of activated T cells (NFAT) is known to regulate PD-1 expression in T cells^35^ and macrophages also express NFATc1,^36, 37^ we therefore wanted to investigate whether NFATc1 is involved in PD-1 expression. We studied the translocation kinetics of NFATc1 in H_2_O_2_-treated RAW 264.7 macrophages and found that nuclear localization of NFATc1 was substantially increased (*P*<0.001) as observed upto 24 h (Figure 6c). This corroborates with the protein level expression of PD-1 after H_2_O_2_ treatment (Figure 1b). Next, to determine whether NFATc1 is involved in PD-1 repression during *L. donovani* infection, nuclear localization of NFATc1 was assessed in infected macrophages, which documented a steady level upto 2 h and started declining only at 4 h post infection (68.9% decrease compared to 2 h infected control, *P*<0.001; Figure 6d). We further validated these findings in BMDM, which also depicted similar findings (Figure 6e). Upon cyclosporine A (NFATc1 inhibitor) treatment, NFATc1 levels in nuclear fractions of infected macrophages were no longer detectable (Figure 6f). Correspondingly, no detectable PD-1 level was obtained in the cyclosporine A-treated infected cells (Figure 6g) further suggesting the importance of NFATc1 in regulation of PD-1 in infection. Similar results were obtained when we used NFATc1 siRNA transfection system to specifically inhibit NFATc1 (Supplementary Figures 4 and 5). The efficiency of NFATc1 knockdown was determined by western blotting which showed 83.6% (Supplementary Figure 6).

## Discussion

In the present study, we showed that macrophage-associated PD-1 contributes to macrophage apoptosis through negatively regulating pro-survival AKT activation. We also elucidated the mechanism involving *L*. *donovani* infection-mediated suppression of PD-1 which in turn, favored sustenance of infection-induced AKT activation thus leading to arrest of macrophage apoptosis and parasite survival.

Increased expression of macrophage-PD-1 in response to apoptotic stimuli H_2_O_2_ along with increased macrophage apoptosis in the presence of PD-1 agonist suggested an apoptosis-inducing role of PD-1. Moreover, our finding that PD-1 exerts antagonistic influence on pro-survival AKT activation is in line with the report that PD-1 pathway negatively regulates LPS-mediated IL-12 production in macrophage.^16^ In the present study, *L. donovani* infection caused downregulation of PD-1 expression along with concomitant activation of AKT. This is in agreement with earlier studies, which showed that *Leishmania* infection resulted in activation of AKT pathway leading to increased intracellular survival of parasites.^20, 21^ Moreover, the observation that prior blockade of PD-1 or PD-IL-1 enhanced the extent of AKT activation, which was reversed by agonist pretreatment corroborated further the antagonistic influence of PD-1 pathway on AKT activation. Furthermore, our findings that PD-1 pathway negatively regulates BAD phosphorylation both in LPS-treated macrophages and in *L. donovani-*infected macrophages suggested the antagonistic influence of PD-1 pathway on proapoptotic BAD inhibition.

We also focused our attention to the mechanistic details pertaining to PD-1 signaling-mediated AKT inactivation. Despite the presence of both ITIM and ITSM in PD-1, recruitment of SHP1 and SHP2 particularly to the ITSM motif of PD-1 is instrumental in mediating inhibitory functions of PD-1.^1, 2^ Furthermore, the fact that PD-1 is capable of suppression of PI3K/AKT activation was mainly due to its ITSM added further importance to this domain in mediating PD-1 signal transduction.^2^ Indeed our co-immunoprecipitation studies supported the notion in providing evidence of stronger association of SHP2 with PD-1, which was markedly inhibited during infection. The role of SHP2 in AKT activation was further supported by the observation that PD-1 agonist-mediated AKT deactivation was significantly reversed in the presence of SHP2 inhibitor. This is in line with the findings of Cho *et al.,*^16^ which showed that PD-1 engagement with B7-H1.Fc fusion protein resulted in the suppression of IL-12 production in macrophages, which was mediated by inhibition of PI3K/AKT pathway through the recruitment of SHP2 to PD-1 cytoplasmic tail.

To get further insight into infection-induced downregulation of PD-1, the role of two transcription factors responsible for PD-1 expression, namely STAT1 and NF-κB were investigated, activation status of which were, however, not changed during the time course of infection. Dampening of NF-κB activation during *L. donovani* infection is well documented,^38, 39^ and STAT1 tyrosine phosphorylation and STAT1α levels were also reported to be decreased in *Leishmania*-infected macrophages.^40^ According to a recent study, NFATc1 is the transcription factor known to regulate PD-1 transcription in T cells.^35^ As NFATc1 is expressed in macrophages and has a role in innate immune regulation,^36, 37^ it therefore seemed likely that NFATc1 might have a role in regulating PD-1 expression even in macrophages. H_2_O_2_ treatment resulted in NFATc1 activation, which is consistent with H_2_O_2_-mediated PD-1 induction, thus suggesting the involvement of NFATc1 in PD-1 induction. Furthermore, *L. donovani* infection dampened the expression and translocation of NFATc1 and this resulted in PD-1 repression. Inhibition of NFATc1 by cyclosporine A or by siRNA-mediated silencing of NFATc1 produced an inhibitory effect on PD-1 expression, thereby suggesting that *L. donovani* infection-mediated PD-1 repression is being regulated by nuclear translocation of NFATc1.

**Figure 7:**
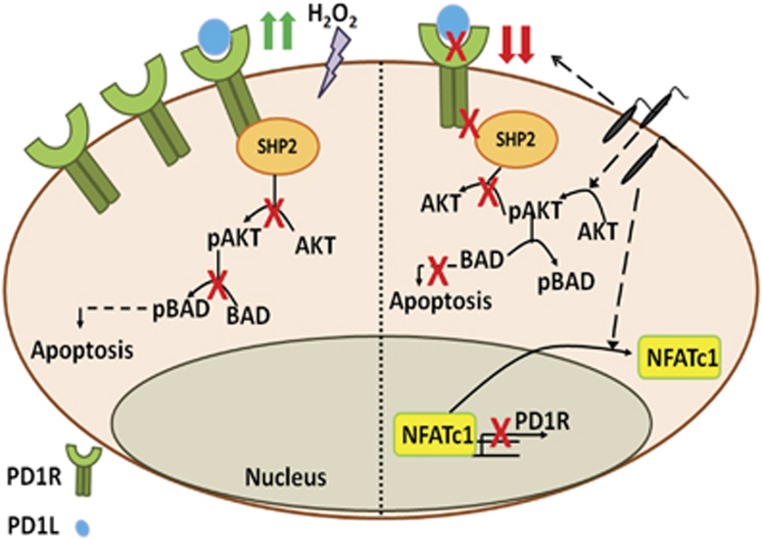
Role of PD-1/SHP2 signaling axis in macrophage apoptosis and parasite survival. Apoptotic stimuli H_2_O_2_ increases the expression of macrophage PD-1 resulting in recruitment of SHP2 at its cytosolic domain. PD-1-associated SHP2 in turn dephosphorylates and inactivates AKT, which could no longer phosphorylate and inactivate proapoptotic BAD, thus leading to macrophage apoptosis. *Leishmania* infection, on the other hand, significantly decreases PD-1 expression level by repressing its transcription factor NFATc1. Reduced PD-1 could no longer recruit SHP2 and therefore AKT is activated and in turn phosphorylates and inactivates BAD thus preventing it to induce apoptosis resulting in parasite survival.

On the whole, the present study not only unravels a relatively novel mechanism of suppression of macrophage apoptosis in *L. donovani* infection, but it also sheds light on the previously unappreciated role of macrophage-associated PD-1 receptor in contributing to macrophage apoptosis. Besides, the study showed that in the context of infection, the death receptor PD-1 remains repressed to facilitate sustenance of infection-induced activation of pro-survival AKT and inhibition of proapoptotic BAD thus favoring parasite survival (Figure 7). This signifies that macrophage PD-1 pertains to be the negative regulator of AKT and probably the reason behind its constitutive expression on macrophages is to tune AKT activation. Based on these results, we may therefore speculate that the inhibition of AKT signaling by induction of PD-1 receptor may serve as future therapeutic tool to regulate macrophage-associated diseases involving the de-regulation of components of the PI3K/AKT pathway.

## Methods

### Chemicals and reagents

Sodium orthovanadate, *Escherichia coli* LPS O111:B4, protease inhibitors, BSA and mouse monoclonal β-actin antibody were obtained from Sigma-Aldrich (St Louis, MO, USA). Penicillin G and streptomycin solution, DMEM, M199, recombinant human GM-CSF and FBS were obtained from Invitrogen (Carlsbad, CA, USA). Antibodies against AKT, p-AKT, STAT1 and p-STAT1 were obtained from Cell Signaling Technology, Danvers, MA, USA. Mouse monoclonal antibodies against PD-1, PD-1L1, NFATc1, SHP2, BAD, p-BAD, protein A/G plus agarose beads and antimouse-AP conjugated secondary antibody were obtained from Santa Cruz Biotechnology (Santa Cruz, CA, USA). BCIP was obtained from Sigma and NBT from Calbiochem (San Diego, CA, USA). Annexin-V-FLUOS staining kit was purchased from Roche Applied Science (Indianapolis, IN, USA). Recombinant PD-1L1-Ig chimera was obtained from R&D Systems (Minneapolis, MN, USA).

### Cell culture, parasites and infection

The promastigotes of *L. donovani* strain (MHOM/IN/1983/AG83) were maintained in Medium 199 (Invitrogen, Life Technologies, Carlsbad, CA, USA) supplemented with 10% fetal calf serum (Invitrogen), 50 μg ml^−1^ streptomycin and 50 U ml^−1^ penicillin. The murine macrophage cell line RAW 264.7 (ATCC) was maintained at 37 °C and 5% CO_2_ in RPMI 1640 (Invitrogen) supplemented with 10% fetal calf serum, streptomycin (100 μg ml^−1^) and penicillin (100 U ml^−1^). BMDM were prepared from the femurs and tibias of 6- to 8-week-old killed BALB/c mice, as described previously.^41^ For *in vitro* infection, macrophages were infected with stationary-phase promastigotes at a 10:1 parasite-to-macrophage ratio for 4 h, non-internalized parasites were removed by washing with medium and the cells were resuspended in medium for an additional 20 h.^42, 43^ The cells were fixed in methanol and stained with Giemsa for determination of the intracellular parasite number.^38^

### Analysis of cell death

Both RAW 264.7 and BMDM (2 × 10^6^) were infected with *L. donovani* promastigotes for different time periods, washed and treated with H_2_O_2_ (400 μm). After 1 h of treatment, the cells were washed with phosphate-buffered saline and incubated overnight at 37 °C and 5% CO_2_. Apoptosis was then determined using annexin-V-FLUOS staining kit (Roche Applied Science) as per the manufacturer’s instructions. The cells were stained with fluorescein isothiocyanate (FITC)-conjugated annexin-V and propidium iodide for 15 min at room temperature in the dark. The stained cells were then analyzed by a FACS Canto IITM cell sorter using 488 nm excitation and 530 nm emissions for FITC and 600 nm for propidium iodide fluorescence using FACS Diva software.

### Surface expression of PD-1 by flow cytometry

Macrophages were infected for different times or treated with H_2_O_2_ (400 μm) for 1 h, washed and fresh media supplemented with FBS was added and kept at 37 °C and 5% CO_2_ for different times. Adherent cells were collected at different time points post treatment to analyze PD-1 expression. The collected cells were incubated with phosphate-buffered saline containing 5% fetal calf serum and 10 μg ml^−1^ murine antibody against PD-1 for 1 h at 4 °C, washed with phosphate-buffered saline three times and then incubated with 10 μg ml^−1^ FITC-conjugated goat antimouse IgG for 30 min at 4 °C. PD-1 expression on the cell surface was analyzed by flow cytometry (FACS). Measurement of fluorescence in cells was made by counting at least 10 000 events per test using a FACS Aria flow cytometer (BD Biosciences, Oxford, UK), and the cells were gated out based on their fluorescent property. Data were analyzed using CellQuest software (BD Biosciences).

### Cytokine analysis by ELISA

The level of various cytokines in the culture supernatants of BMDM were measured using a sandwich ELISA kit (Quantikine M, R&D Systems) as per the instructions of the manufacturer.

### Immunoprecipitation and immunoblotting

Cells were lysed in lysis buffer (Cell Signaling Technology) and the protein concentrations in the cleared supernatants were estimated using a protein assay (Bio-Rad, Hercules, CA, USA). Briefly, pre-cleared cell lysates (500 μg) were incubated overnight with specific primary antibody at 4 °C. For co-immunoprecipitation studies, 25 μl of protein A/G plus agarose beads (Santa Cruz Biotechnology) were added to the mixture and incubated for 4 h at 4 °C. Immune complexes were collected and washed three times with ice-cold lysis buffer and once with lysis buffer without Triton X-100. The immunoprecipitated samples and cell lysates were resolved by 10% SDS-PAGE and then transferred to nitrocellulose membrane (GE Healthcare, Little Chalfont, UK). Thirty micrograms of protein from the whole-cell lysate of each sample were loaded as input. The membranes were blocked with 5% BSA in wash buffer (TBS, 0.1% Tween 20) for 2 h at room temperature and probed with primary antibody overnight at dilution recommended by the suppliers. The membranes were washed three times with wash buffer and then incubated with alkaline phosphatase-conjugated secondary antibody and detected by hydrolysis of 5 bromo-4-chloro-3′-indolylphosphate chromogenic substrate.

### Isolation of nuclear fraction

To prepare subcellular fractions, the cells were lysed by a 10 min hypotonic treatment on ice in buffer A (10 mm HEPES (pH 7.9), 10 mm KCI, 1.5 mm MgCl_2_, 0.5 mm DTT, 0.5 mm phenylmethylsulfonyl fluoride, 10 μg of leupeptin per ml, 10 μg of pepstatin per ml, 0.01 U of aprotinin per ml) followed by homogenization using a narrow gauge syringe. The extract was then centrifuged at 4 °C for 10 min at 10 000 *g*. The supernatant was used as the cytosolic extract. The pellet was washed once with ice-cold buffer A and resuspended in two volumes of buffer C (20 mm HEPES (pH 7.9), 0.42 m NaCl, 1.5 mm MgCl_2_, 0.2 mm EDTA, 0.5 mm dithiothreitol (DTT), 0.5 mm phenylmethylsulfonyl fluoride, 10 μg of leupeptin per ml, 10 μg of pepstatin per ml, 0.01 U of aprotinin per ml, 25% glycerol). After the concentration of NaCl was adjusted to 0.38 m, the suspension was placed at −70 °C for 10 min, thawed slowly on ice and then incubated for 10 min in ice with intermittent tapping. After a 15 min centrifugation at 10 000 *g* at 4 °C, the supernatant solution representing the soluble nuclear fraction was removed.

### Real-time PCR

Total RNA from macrophages was isolated using the RNeasy mini kit (Qiagen, Valencia, CA, USA) according to the manufacturer’s instructions. One microgram of RNA was used as template for cDNA synthesis. Quantitative real-time PCR (ABI 7500 Fast Real Time PCR system, Applied Biosystems) were performed using SYBR green PCR master mix (Applied Biosystems, Foster City, CA, USA). The ABI 7500 Fast Sequence detector was programmed with the following PCR amplification conditions: 40 cycles of 95 °C for 15 s and 60 °C for 1 min. β-Actin was chosen as an internal control for variability in amplification because of differences in initial mRNA concentrations. Relative quantitation was performed using the comparative ΔΔCt method, and data were normalized to β-actin mRNA levels and expressed as a fold change compared with uninfected controls. Primers used are PD-1: sense, 5′-CCAGCAACCAGACTGAAAAAC-3′, antisense, 5′-TCTCCTCGATTTTTGCCTTG-3′, β-actin: sense, 5′-GGCGGACTGTTACTGAGCTG-3′, antisense, 5′-TGCTCCAACCAACTGCTGTC-3′

### siRNA transfection

RAW 264.7 cells (2 × 10^6^) were transfected with 1 μg of either SHP2 or NFATc1 siRNA according to the manufacturer’s instructions (Santa Cruz Biotechnology). Control siRNA containing scrambled sequence was used as negative control. Following silencing, the cells were infected with *L. donovani* promastigotes as described before.

### Densitometric analysis

Densitometric analyses for all the experiments were carried out using QUANTITY ONE software (Bio-Rad). Band intensities were quantified densitometrically, and the values were normalized to endogenous control and expressed in arbitrary units. The ratios of optical density of particular bands/endogenous control are indicated as bar graphs adjacent to figures.

### Statistical analysis

Data shown are representative of at least three independent experiments unless otherwise stated as *n* values given in the legend. Macrophage cultures were set in triplicate, and the results are expressed as the mean±s.d. Student’s *t*-test was used to assess the statistical significance of differences among a pair of data sets with a *P*-value <0.05 considered to be significant.
