# Supplementary material for: The role of PD-1 in regulation of macrophage apoptosis and its subversion by Leishmania donovani
Source: Clin Transl Immunology. 2017 May 5;6(5):e137–. doi: 10.1038/cti.2017.12 (PMC5493582; doi:10.1038/cti.2017.12)
Supplement: Supplementary Information [file cti201712x1.pdf]

Supplementary figures

S1

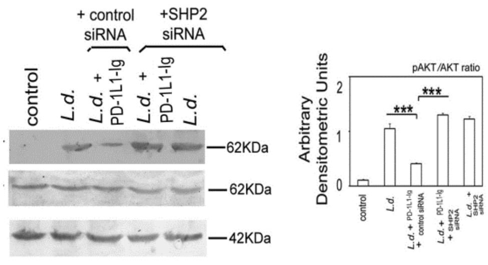

Figure Legend S1:

SHP2 siRNA or control siRNA transfected macrophages were pre-treated with or without PD-1 agonist and infected with *L. donovani* promastigotes for 6 h. Cell were analyzed for p- AKT and AKT levels by Western blot analysis. The data shown are mean ± S.D. of three independent experiments. n=3, ***p < 0.001; Student's t test.

S2

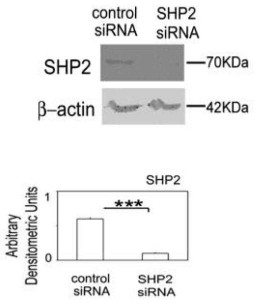

Figure Legend S2:

Expression of SHP2 was analysed in SHP2 siRNA or control siRNA transfected macrophages by immunoblotting. The data shown are mean ± S.D. of three independent experiments. n=3, ***p < 0.001; Student's t test.

S3

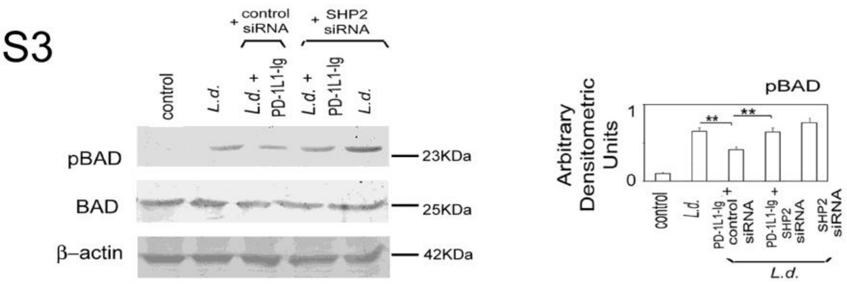

Figure Legend S3:

SHP2 siRNA or control siRNA transfected macrophages were pre-treated with PD-1 agonist and infected with *L. donovani* promastigotes for 6 h. Cell lysates were analyzed for p-BAD and BAD. The data shown are mean ± S.D. of three independent experiments. n=3, **p < 0.01; ***p < 0.001; Student’s t test.

S4

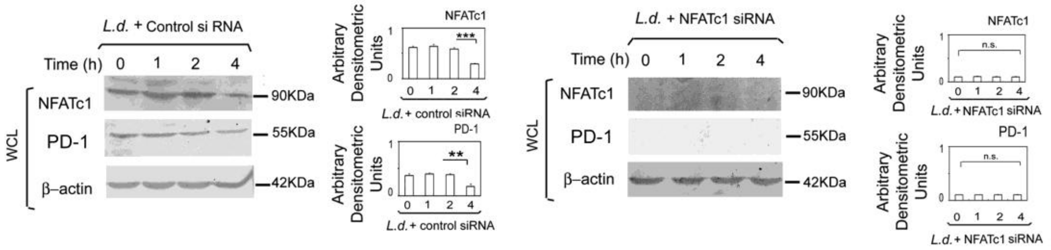

Figure Legend S4:

NFATcl siRNA or control siRNA transfected macrophages were infected with *L. donovani* promastigotes for indicated times and NFATcl and PD-1 levels were analysed in whole cell lysates. The data shown are mean ± S.D. of three independent experiments. n=3, n.s. non-significant; **p < 0.01; ***p < 0.001; Student’s t test.

S5

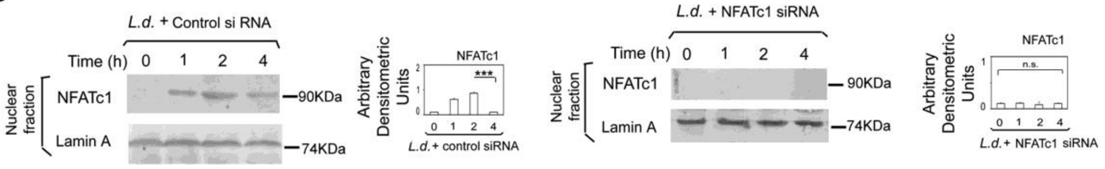

Figure Legend S5:

NFATcl siRNA or control siRNA transfected macrophages were infected with *L. donovani* promastigotes for indicated times. Cells were lysed, nuclear fractions were prepared and NFATcl levels were analysed in nuclear fractions by western blotting. The data shown are mean ± S.D. of three independent experiments. n=3, n.s. non-significant; ***p < 0.001;

Student’s t test.

S6

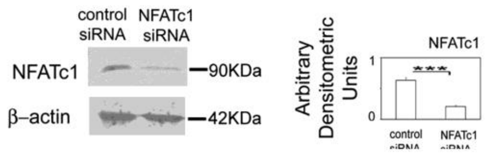

Figure Legend S6:

Expression of NFATcl was analysed in NFATcl siRNA or control siRNA transfected macrophages by immunoblotting. The data shown are mean ± S.D. of three independent experiments. n=3, ***p < 0.001; Student’s t test.
